# Li_2_ZrO_3_-Coated Monocrystalline LiAl_0.06_Mn_1.94_O_4_ Particles as Cathode Materials for Lithium-Ion Batteries

**DOI:** 10.3390/nano11123223

**Published:** 2021-11-27

**Authors:** Chunliu Li, Banglei Zhao, Junfeng Yang, Linchao Zhang, Qianfeng Fang, Xianping Wang

**Affiliations:** 1 Key Laboratory of Materials Physics, Institute of Solid State Physics, HFIPS, Chinese Academy of Sciences, Hefei 230031, China; lcliu@southmn.com (C.L.); qffang@issp.ac.cn (Q.F.); 2 Science Island Branch, Graduate School of University of Science and Technology of China, Hefei 230026, China; 3 School of Management Science and Engineering, Anhui University of Finance and Economics, Bengbu 233030, China; zbl1234@mail.ustc.edu.cn; 4 Lu’an Branch, Anhui Institute of Innovation for Industrial Technology, Lu’an 237100, China

**Keywords:** lithium zirconium oxide, lithium manganese oxide, monocrystallite, cathode materials, lithium-ion batteries

## Abstract

Li_2_ZrO_3_-coated and Al-doped micro-sized monocrystalline LiMn_2_O_4_ powder is synthesized through solid-state reaction, and the electrochemical performance is investigated as cathode materials for lithium-ion batteries. It is found that Li_2_ZrO_3_-coated LiAl_0.06_Mn_1.94_O_4_ delivers a discharge capacity of 110.90 mAhg^−1^ with 94% capacity retention after 200 cycles at room temperature and a discharge capacity of 104.4 mAhg^−1^ with a capacity retention of 87.8% after 100 cycles at 55 °C. Moreover, Li_2_ZrO_3_-coated LiAl_0.06_Mn_1.94_O_4_ could retain 87.5% of its initial capacity at 5C rate. This superior cycling and rate performance can be greatly contributed to the synergistic effect of Al-doping and Li_2_ZrO_3_-coating.

## 1. Introduction

Spinel LiMn_2_O_4_ (LMO) has been recognized as one of the promising cathode materials for the lithium-ion batteries due to the environmental compatibility, low cost, and high specific capacity [1,2,3,4]. However, the poor rate performance caused by the low Li^+^ conductivity and the rapid capacity fading induced by the Jahn–Teller distortion have severely impeded its commercial applications [5,6,7].

Nanocrystallization has been proved to be an effective way to improve the electrochemical kinetics by decreasing the diffusion path of Li^+^ and enlarging contact area between electrode and electrolyte [8,9]. Nevertheless, the enlarged specific surface area will inevitably increase the side reactions. Although the ion-doping technique could alter the electrochemical properties to some extent, the capacity decay still occurs due to the Mn ions dissolution, especially under high temperatures [10,11,12,13]. Alternatively, surface coating is often adopted to improve the cycling performance by inhibiting the side reactions between LMO and electrolyte [14,15]. Unfortunately, the coating strategy cannot improve the transport kinetics of Li^+^. Therefore, the combination of ion-doping and coating should be considered to make full advantages.

Except for the side reactions, the nanocrystallization will lead to flawed crystallinity which is harmful for the Li^+^ ion transport, however LMO has a unique three-dimensional tunnel structure in which the Li^+^ could transfer through the 8a-16c-8a pathway [16]. Therefore, the monocrystal size should not be the determining factor to restrain the Li^+^ transport. Moreover, the micron-sized monocrystalline LMO modified with ion-doping and surface coating may simultaneously decrease the contact area with electrolyte and improve the electrochemical performance.

In this work, we propose combined tactics to fabricate micro-sized monocrystalline LiAl_0.06_Mn_1.94_O_4_ particles with Li_3_BO_3_ as sintering additive. The resultant Li_2_ZrO_3_-coated and Al-doped micro-sized monocrystalline LiMn_2_O_4_ powder was adopted as cathode materials for lithium-ion batteries, and their electrochemical performance was investigated in detail.

## 2. Materials and Methods

The Li_2_ZrO_3_-coated micro-sized monocrystalline LiAl_0.06_Mn_1.94_O_4_ was prepared with solid-state sintering. First, Li_2_CO_3_ (Aldrich, St. Louis, MI, USA), Al_2_O_3_ (Aldrich) and Mn_3_O_4_ (EMD, Aldrich) were ball-milled with the molar ratio of Li:Al:Mn = 1.06:0.06:1.94. The excess Li_2_CO_3_ was used to compensate the lithium loss during heat treating. Then, the mixture was sintered at 450 °C for 5 h. After that, the pre-sintered mixture was ball-milled with Li_2_CO_3_, H_3_BO_3_, and ZrO_2_. Then, the mixture was sintered at 780 °C for 18 h. Li_3_BO_3_ and Li_2_ZrO_3_ could be synthesized as the following Equations (1) and (2).

H_3_BO_3_ + Li_2_CO_3_ → Li_3_BO_3_ (1)

ZrO_2_ + Li_2_CO_3_ → Li_2_ZrO_3_ (2)

The detailed ingredient of the micron-sized monocrystalline LiAl_0.06_Mn_1.94_O_4_ was listed in Table 1. As a comparison, the LiMn_2_O_4_ samples without Al-doping, Li_3_BO_3_ additive or Li_2_ZrO_3_-coating were also prepared through the same sintering process.

Structural characterization of the samples was analyzed by X-ray diffraction (XRD, Rigaku SmartLab diffractometer) with Cu Kα radiation in the 2θ range of 10–70°. The particle size, surface morphology, and elemental composition were observed by scanning electron microscopy (SEM, Hitachi S-3400N) equipped with an energy dispersive spectrometer (EDS, Oxford 7426). The microstructure was detected with transmission electron microscopy (TEM, FEI Tecnai G2 F20). The samples were smashed before the TEM analysis.

Synthesized LiMn_2_O_4_ particle was mixed with carbon black and polyvinylidene fluoride (PVDF) with the mass ratio of 90:5:5 in N-methyl pyrrolidinone (NMP). Then, the slurry was coated on the aluminum foil and vacuum-dried at 100 °C. The electrode laminate was roll-pressed and punched to be discs with a diameter of 14 mm. The loading density of active material is approximately 5–8 mg cm^−2^. The electrolyte was 1 M LiPF_6_ in dimethyl carbonate (DMC)/ethyl carbonate (EMC)/ethylene carbonate (EC) (1:1:1, *v*/*v*/*v*). The 2032-type coin cells using Li metal as anode were prepared in an argon-filled glove box and tested in the voltage range of 3.0–4.3 V at several rates (1C = 148 mAhg^−1^) on a battery test system (Neware). The cells were charged/discharged at 0.2C for 3 times first and then cycled at 0.5C for 200 times at 25 °C. Meanwhile, same cells were charged/discharged at 0.2C for 3 times and at 0.5C for 100 times at 55 °C, successively. Moreover, to evaluate the rate performance, the cells were also charged at 0.5C and discharged at 0.5C, 1C, 2C, 3C, and 5C, respectively.

The electrochemical impedance spectroscopy (EIS) of the cells was performed on electrochemical workstation (CHI600E) with the frequency range of 10^5^ to 10^−2^ Hz. The EIS tests were performed after the 1st and 200th cycle, respectively.

## 3. Results and Discussion

### 3.1. Li_3_BO_3_ Additive Promote the Grain Growth

**Figure 1:**
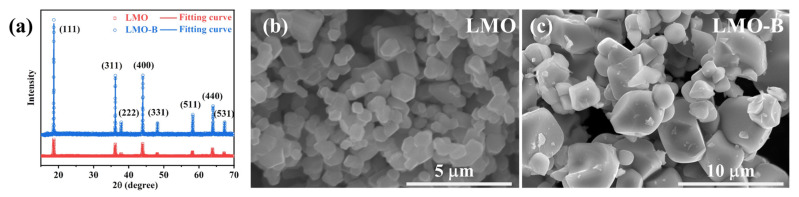
(**a**) XRD patterns of the LMO and LMO-B samples; the SEM images of the (**b**) LMO and (**c**) LMO-B.

The XRD patterns of the samples are shown in Figure S1. All the diffraction peaks match well with LiMn_2_O_4_ (JCPDF 35-0782). It proves that the Al-doping Li_3_BO_3_ additive or the Li_2_ZrO_3_-coating has no effect on the spinel cubic structure with the spatial group Fd3m. While focusing on the effect of the Li_3_BO_3_ additive, it could be found that the full width at half maximum (FWHM) of the LMO-B is much broader than that of the LMO (Table S1). As shown in Figure 1a, the FWHM of the main peaks of LMO are 0.188 (111), 0.152 (311), 0.155 (222), 0.163 (400), 0.151 (331), and 0.176 (511), respectively. While the corresponding values for the LMO-B are 0.071, 0.061, 0.053, 0.064, 0.066, and 0.073, respectively. It is suggested that the grain size of the monocrystal LiMn_2_O_4_ has grown up with the help of the Li_3_BO_3_ additive. Meanwhile, the conclusion is verified by the morphological observation (Figure 1b,c). The grain size of the LMO is less than 2 μm, while that of the LMO-B sample is approximately 2–8 μm according to the particle size distribution analysis (Figure S2). Both the XRD and SEM results prove that the Li_3_BO_3_ additive could promote the grain growth of monocrystal LiMn_2_O_4_ effectively.

### 3.2. Effect of the Al-Doping

**Figure 2:**
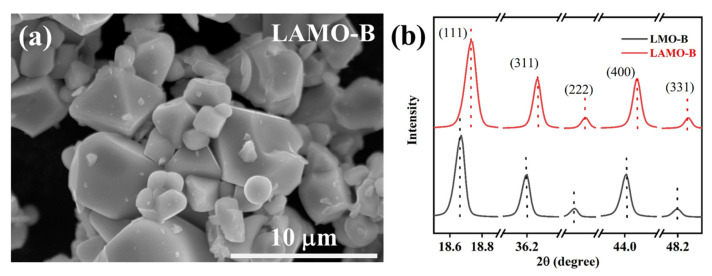
(**a**) Morphology of the LAMO-B sample. (**b**) The comparison of diffraction peaks of LMO-B and LAMO-B samples.

With the Al-doping in LiMn_2_O_4_, the morphology of the LAMO-B (Figure 2a) has no significate change in comparison with the LMO-B sample (Figure 1c). While the systematic right shift of diffraction peaks could be observed, compared with the LMO-B sample, the main diffraction peaks of the LAMO-B sample shifted toward to the high angles as shown in Figure 2b. Meanwhile, the calculated lattice parameter of the LMO-B and LAMO-B is 8.2235 and 8.2090, respectively. It means that the Al atom with smaller radius has occupied the Mn site in the monocrystal LiMn_2_O_4_ lattice structure.

### 3.3. Li_2_ZrO_3_-Coating on the Monocrystal LiMn_2_O_4_

**Figure 3:**
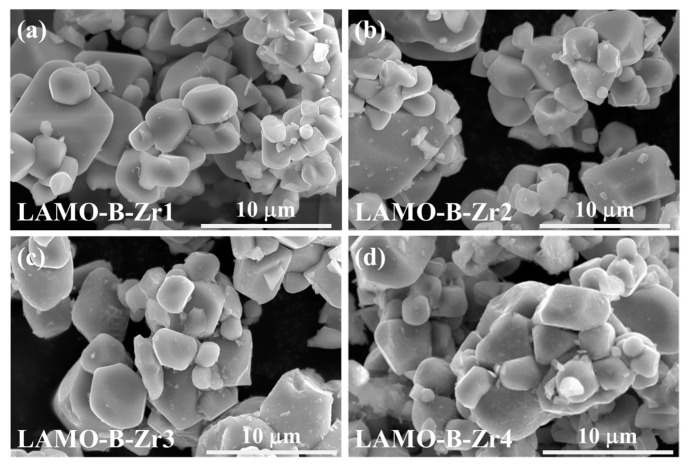
The SEM images of the (**a**) LAMO-B-Zr1; (**b**) LAMO-B-Zr2; (**c**) LAMO-B-Zr3 and (**d**) LAMO-B-Zr4 samples.

To analyze the influence of the Li_2_ZrO_3_-coating amount, the morphologies of the four samples with Al-doping and Li_2_ZrO_3_ coating were compared as shown in Figure 3. It is found that the micro-sized large grains are surrounded by smaller ones in all four samples and all the secondary particles show a diameter as large as ~10 mm. It proves that the Li_2_ZrO_3_ coating could not affect the size distribution.

**Figure 4:**
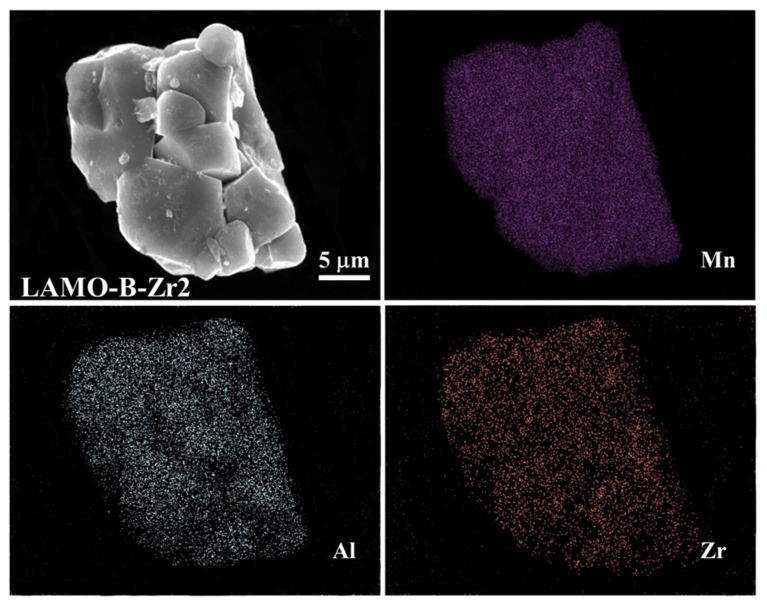
The EDS mapping of the LAMO-B-Zr2 sample.

Meanwhile, it is found that the Al-doping and Li_2_ZrO_3_ coating distribute uniformly in the final samples. Taking the LAMO-B-Zr2 sample for example, the EDS mapping results exhibit uniform distribution of the Mn, Al, and Zr elements in the secondary particles as shown in Figure 4.

**Figure 5:**
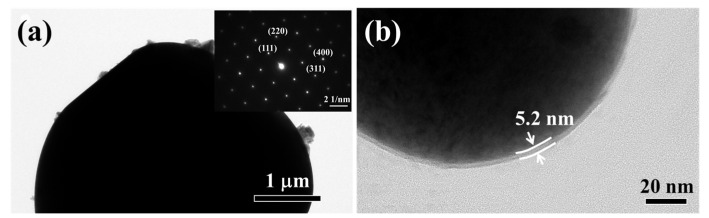
The TEM images and corresponding selected area electron diffraction (SAED) of the (**a**) LMO-B and (**b**) LAMO-B-Zr2.

To further observe the microstructure of the samples, TEM analysis and corresponding selected area electron diffraction (SAED) of the LMO-B are shown in Figure 5a. The diffraction spots can be indexed to (220), (111), (311), and (400) planes of cubic spinel LiMn_2_O_4_ (JCPDS 35-0782). Compared with the pristine surface of the LMO-B sample, a coating layer with a thickness of ~5.2 nm could be found in the LAMO-B-Zr2 sample (Figure 5b). Moreover, the coating layer should be brought from the Li_2_ZrO_3_ additive.

### 3.4. Electrochemical Performances

**Figure 6:**
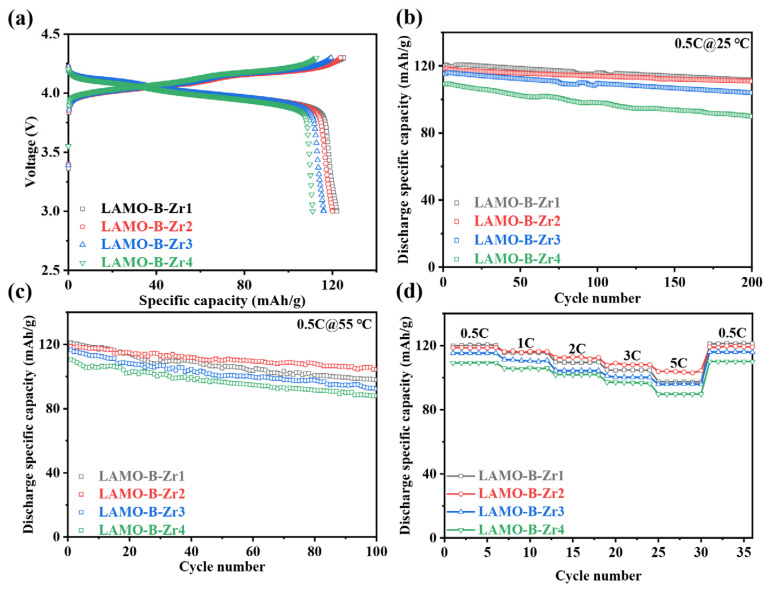
The electrochemical performance of the Li_2_ZrO_3_-coated LAMO-B-Zr cells. (**a**) The initial charge–discharge profiles at 0.2C and 25 °C; The cycling performance with 0.5C rate at (**b**) 25 °C and (**c**) 55 °C. (**d**) The rate performance at 0.5C, 1C, 2C, 3C and 5C, respectively.

The cycling and rating performance of the cells with the Al-doping and Li_2_ZrO_3_-coating samples was tested at the same conditions (Figure 6). The initial discharge capacity of the cells at 25 °C and 0.2C rate decreased steadily with the increscent Li_2_ZrO_3_-coating as shown in Figure 6a. It is reasonable, as the Li_2_ZrO_3_ provides no capacity contribution, the more coating, the less proportion of the active materials in the cell. Nevertheless, Li_2_ZrO_3_ aimed at reducing the side reactions serving as a protect layer. The effect of the Li_2_ZrO_3_-coating amount on the cycling performance at 25 °C and 55 °C is shown in Figure 6b,c, respectively. It was found that the capacity retention of the four samples is 93.0%, 93.9%, 90.2%, and 82.3% at 25 °C after 200 cycles with the increasing Li_2_ZrO_3_-coating amount. Moreover, at 55 °C, the corresponding capacity retention is 81.0%, 87.5%, 79.2%, and 79.5%, respectively. It is obvious that the LAMO-B-Zr2 could decrease the capacity loss and provide the optimal protection at both temperatures. Moreover, as shown in Figure 6d, the rate performance of LAMO-B-Zr2 is also better than the other three samples, especially at higher rates. Although the discharge capacity of LAMO-B-Zr2 at 0.5C is less than the LAMO-B-Zr1, the capacity at 1, 2, 3, and 5C rates is larger than the other three samples. It proves that the deficiency of Li_2_ZrO_3_ could not provide overall protection from the side reactions, while the excess of Li_2_ZrO_3_ would impede the migration of lithium ion. LAMO-B-Zr2 exhibited the best performance in a series of Al-doping and Li_2_ZrO_3_ coating samples.

**Figure 7:**
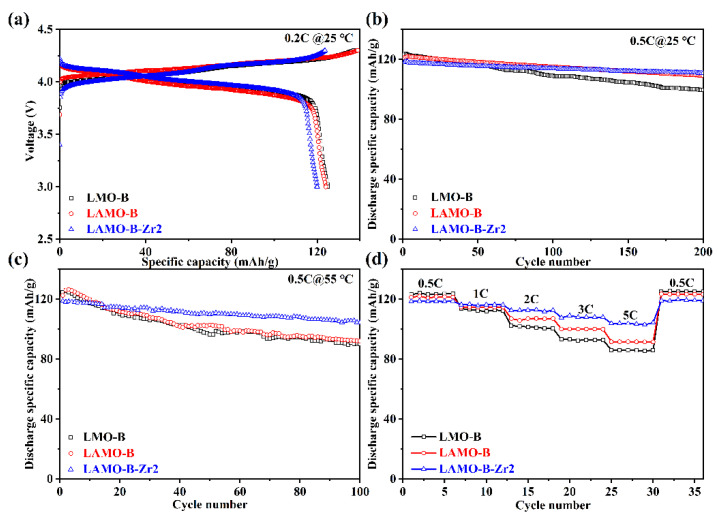
The electrochemical performance of the LMO-B, LAMO-B, and LAMO-B-Zr2 cells. (**a**) The initial charge-discharge profiles at 0.2 °C and 25 °C. The cycling stability with 0.5C rate at (**b**) 25 °C and (**c**) 55 °C. (**d**) The rate performance at 0.5C, 1C, 2C, 3C, and 5C, respectively.

To explore the influence of the Al-doping and the Li_2_ZrO_3_ coating, the electrochemical performance of the LMO-B, LAMO-B, and LAMO-B-Zr2 samples were characterized in parallel. Figure 7a presents the first charge–discharge curves obtained at 0.2C and 25 °C between 3.0 and 4.3 V (vs. Li/Li^+^). There are two voltage plateaus near 4.0 V and 4.1 V, which indicates the two-stage processes of Li^+^ ion insertion/extraction reactions for a typical characterization of a well-crystallized spinel LiMn_2_O_4_ [17,18]. The initial discharge specific capacities of the as-prepared samples show a decline trend with the Al-doping and the Li_2_ZrO_3_ coating. This result is comprehensible because the percentage of Mn^3+^ is partially occupied by the Al-doping or lowered by the Li_2_ZrO_3_ coating.

However, according to the cycling stability at room temperature and 55 °C shown in Figure 7b,c, respectively, the capacity retention of the LAMO-B-Zr2 is much higher than that of the LMO-B and LAMO-B (Table 2). The possible reason may lie in that the Al-doping could stable the crystal structure during the cycling. Analogous results could be found in other literature reports [19,20]. On the other hand, with the help of the Li_2_ZrO_3_ coating, the capacity retention rate of the LAMO-B-Zr2 could go further to 93.9% after 200 cycles at 25 °C and keep at 87.8% after 100 cycles at 55 °C. It turns out that the Li_2_ZrO_3_-coating could suppress the side-reactions effectively.

Otherwise, the rate performance of the cells between 3.0 and 4.3 V (vs. Li/Li^+^) is shown in Figure 7d. All the cells exhibit reduced discharge capacities as the increased C-rates. Although the capacity of LAMO-B-Zr2 at 0.5C is inferior to the other cells as the lowered proportion of active substances, the discharge capacities themselves or the capacity retention of the LAMO-B-Zr2 at 1C, 2C, 3C, and 5C rates are much higher than the other control samples. Moreover, the discharge capacity of LAMO-B-Zr2 could reach up to 103.41 mAhg^−1^ at 5C.

The specific discharge capacity and the capacity retention of the LAMO-B-Zr2 sample at both room temperature and 55 °C are better than those of some reported LiMn_2_O_4_ single-crystalline and Al-doped LiMn_2_O_4_. The detailed comparisons of the electrochemical performance are listed in Table S2.

**Figure 8:**
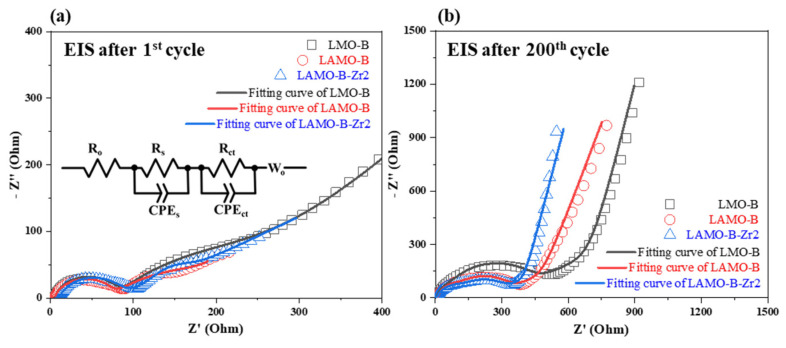
The Nyquist plots of the LMO-B, LAMO-B, and LAMO-B-Zr2 cells after the (**a**) 1st and (**b**) 200th cycle over the frequency range of 10^5^ to 10^−2^ Hz. Inset figure is the equivalent circuit.

Furthermore, the EIS results of the LMO-B, LAMO-B, and LAMO-B-Zr2 cells after the 1st and 200th cycle is shown in Figure 8a,b, respectively. All the cells are discharged to 4.0 V before testing. All the Nyquist plots comprises two depressed semicircles in the high and middle frequency region and a straight line in the low frequency region. The intersection at the high frequency shows the Ohmic resistance (R_o_), the diameter of the semicircle at the high frequency means the resistance (R_s_) of solid electrolyte interface (SEI), and the diameter of the semicircle at low frequency suggests the resistance of the charge transfer (R_ct_). The right intersection of the semicircle with the horizontal axis in the low frequency represent the total internal resistance (R_t_ = R_o_ + R_s_ + R_ct_) [21,22]. The EIS plots were fitted with equivalent circuit shown in Figure 8a by Zview software. Moreover, the corresponding fitting results of the impedance are listed in Table 3. It is found that the Al-doping could decrease the impedance compared with the pristine LMO-B and the LAMO-B samples. It is suggested that the coating strategy is an efficient method of decreasing impedance.

## 4. Conclusions

The micron-sized monocrystalline LiAl_0.06_Mn_1.94_O_4_ with a grain size of 2–8 μm was prepared in this work. With the synergistic modification of Al-doping and Li_2_ZrO_3_ coating, the cycling stability and rating performance have been significantly improved. Meanwhile, the optimal Li_2_ZrO_3_ coating amount was investigated. The LAMO-B-Zr2 with 2 mol% Li_2_ZrO_3_ addition possesses capacity retention of 93.9% after 200 cycles at room temperature and 87.8% after 100 cycles at 55 °C. This work provides appreciable point of view into the practical application of the LMO cathode in lithium-ion batteries.

## Figures and Tables

**Table 1:** Specific component of the micron-sized monocrystalline LiMn_2_O_4_ samples (* No addition).

Samples	Composition		
	LiMn_2_O_4_	Li_3_BO_3_	Li_2_ZrO_3_
LMO	LiMn_2_O_4_	*	*
LMO-B	LiMn_2_O_4_	1 mol%	*
LAMO-B	Li Al_0.06_Mn_1.94_O_4_	1 mol%	*
LAMO-B-Zr1	Li Al_0.06_Mn_1.94_O_4_	1 mol%	1 mol%
LAMO-B-Zr2	Li Al_0.06_Mn_1.94_O_4_	1 mol%	2 mol%
LAMO-B-Zr3	Li Al_0.06_Mn_1.94_O_4_	1 mol%	3 mol%
LAMO-B-Zr4	Li Al_0.06_Mn_1.94_O_4_	1 mol%	4 mol%

**Table 2:** The capacity retention ratios of LMO-B, LAMO-B, and LAMO-B-Zr2.

Samples	Capacity Retention Ratios	
	After 200 Cycles at 25 °C	After 100 Cycles at 55 °C
LMO-B	81.0%	72.2%
LAMO-B	90.0%	75.2%
LAMO-B-Zr2	93.9%	87.8%

**Table 3:** The fitting results of the LMO-B, LAMO-B, and LAMO-B-Zr2 cells after the 1st and 200th cycle.

Sample	Rt (Ω) after 1st Cycle	Rt (Ω) after 200th Cycle
LMO-B	248.1	377.8
LAMO-B	235.8	293.1
LAMO-B-Zr2	101.1	270.8

## Data Availability

Data presented in this study are available on request from the corresponding authors.

## Supplementary Materials

The following are available online at https://www.mdpi.com/article/10.3390/nano11123223/s1, Figure S1: The XRD patterns of the LiMn_2_O_4_ samples; Figure S2. The particle size distribution of the (**a**) LMO; (**b**) LMO-B; (**c**) LAMO-B-Zr1; (**d**) LA-MO-B-Zr2; (**e**) LAMO-B-Zr3; (**f**) LAMO-B-Zr4; Table S1: The full width at half maximum (FWHM) of the LMO and LMO-B samples; Table S2: Comparison of electrochemical performance of different LiMn_2_O_4_ electrode materials.
