# Supplementary material for: Li2ZrO3-Coated Monocrystalline LiAl0.06Mn1.94O4 Particles as Cathode Materials for Lithium-Ion Batteries
Source: Nanomaterials (Basel). 2021 Nov 27;11(12):3223. doi: 10.3390/nano11123223 (PMC8709395; doi:10.3390/nano11123223)
Supplement: Supplementary file 1 [file nanomaterials-11-03223-s001.zip › nanomaterials-1451868-supplementary.pdf]

nanomaterials

Supplementary Materials

Li_2ZrO_3-Coated Monocrystalline $LiAl_{0.06}Mn_{1.94}O_4$ Particles as Cathode Materials for Lithium-Ion Batteries

Chunliu Li [1,2,†], Banglei Zhao [3,†], Junfeng Yang [1,4,*], Linchao Zhang [1,*], Qianfeng Fang [1,2] and Xianping Wang [1,*]

[1] Key Laboratory of Materials Physics, Institute of Solid State Physics, HFIPS, Chinese Academy of Sciences, Hefei 230031, China; lcliu@southmn.com (C.L.); qffang@issp.ac.cn (Q.F.)
[2] Science Island Branch, Graduate School of University of Science and Technology of China, Hefei 230026, China
[3] School of Management Science and Engineering, Anhui University of Finance and Economics, Bengbu 233030, China; zbl1234@mail.ustc.edu.cn
[4] Lu'an Branch, Anhui Institute of Innovation for Industrial Technology, Lu'an 237100, China
* Correspondence: jfyang@issp.ac.cn (J.Y.); lczhang@issp.ac.cn (L.Z.); xpwang@issp.ac.cn (X.W.)
† These authors contributed equally to this work.

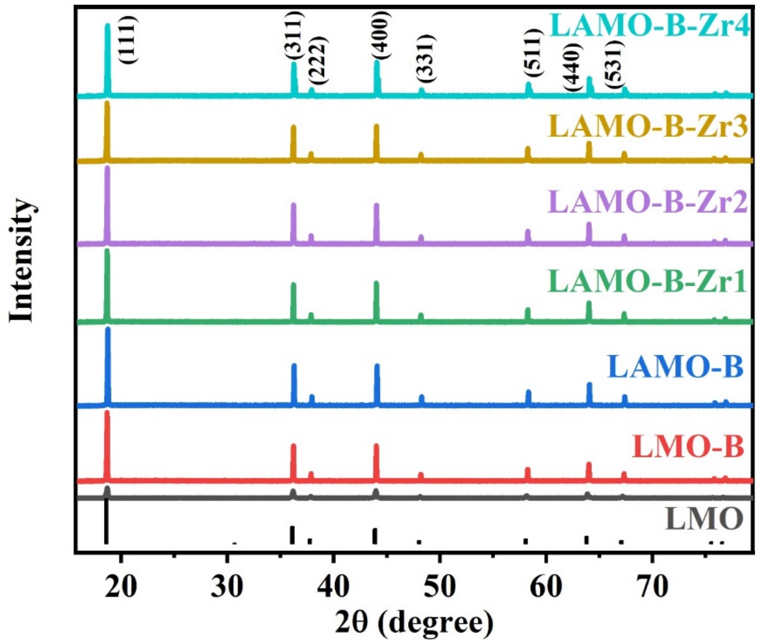

Figure S1. The XRD patterns of the $LiMn_2O_4$ samples.

Table S1. The full width at half maximum (FWHM) of the LMO and LMO-B samples.

	**2θ**	**(hkl)**	**FWHM**
**LMO**	18.69	111	0.188
	36.17	311	0.152
	37.84	222	0.155
	43.96	400	0.163
	48.12	331	0.151
	58.16	511	0.176
	**2θ**	**(hkl)**	**FWHM**
**LMO-B**	18.67	111	0.071
	36.20	311	0.061
	37.87	222	0.053
	44.01	400	0.064
	48.20	331	0.066
	58.26	511	0.073

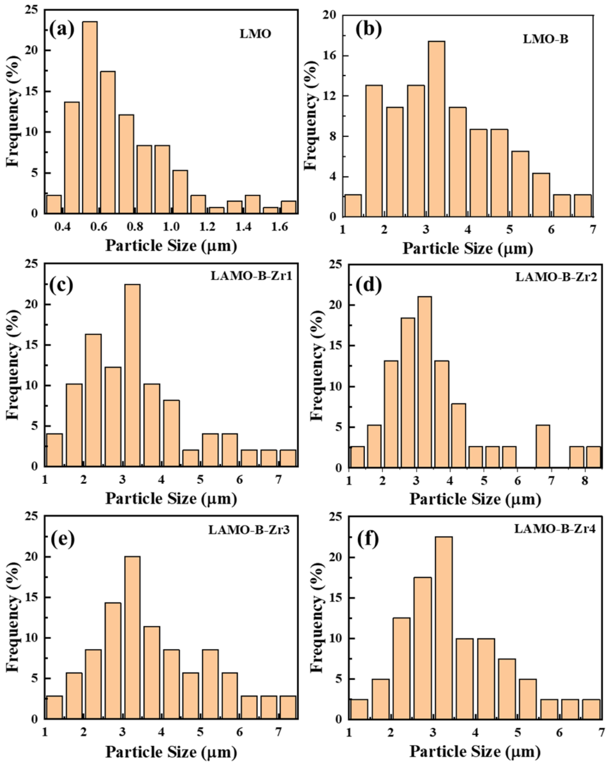

Figure S2. The particle size distribution of the (**a**) LMO; (**b**) LMO-B; (**c**) LAMO-B-Zr1; (**d**) LAMO-B-Zr2; (**e**) LAMO-B-Zr3; (**f**) LAMO-B-Zr4.

Table S2. Comparison of electrochemical performance of different $LiMn_2O_4$ electrode materials.

Electrode material	Discharge Capacity (mAh/g)	Capacity retention	Reference
LAMO-B-Zr2	118.88@0.5C & 55 °C<br>104@5C & 25 °C	94% after 200 cycles@0.2C & 25 °C<br>87.8% after 100 cycles@0.2C & 55 °C	This work
$LiAl_{0.1}Mn_{1.9}O_4$<br>Single-Crystalline Nanotubes	110@0.1C & 55 °C<br>90@5C & 25 °C	80% after 200 cycles@5C & 55 °C	[1]
$LiAl_{0.1}Mn_{1.9}O_4$	90@2C & 55 °C<br>106@5C & 25 °C	88.7% after 1000 cycles@2C & 25 °C<br>86.1% after 500 cycles@2C &55 °C	[2]
Single-crystalline $LiMn_2O_4$ nano-tubes	80@5C & 25 °C	70% after 1500 cycles @5C & 25 °C	[3]

References

1. Ding, Y.L.; Xie, J.; Cao, G.S.; Zhu, T.J.; Yu, H.M.; Zhao, X.B. Enhanced elevated-temperature performance of Al-doped single-crystalline $LiMn_2O_4$ nanotubes as cathodes for lithium ion batteries. *J. Phys. Chem. C* **2011**, *115*, 9821–9825.
2. Fu, Y.; Jiang, H.; Hu, Y.; Dai, Y.; Zhang, L.; Li, C. Synergistic enhancement effect of Al doping and highly active facets of $LiMn_2O_4$ cathode materials for lithium-ion batteries. *Ind. Eng. Chem. Res.* **2015**, *54*, 3800–3805.
3. Ding, Y.L.; Xie, J.; Cao, G.S.; Zhu, T.J.; Yu, H.M.; Zhao, X.B. Single-crystalline $LiMn_2O_4$ nanotubes synthesized via template-engaged reaction as cathodes for high-power lithium ion batteries. *Adv. Funct. Mater.* **2011**, *21*, 348–355.
